# Quantitative analysis of robustness‐based versus LET‐based optimization in intensity‐modulated proton therapy for pediatric brain tumors

**DOI:** 10.1002/acm2.70472

**Published:** 2026-02-18

**Authors:** Fariha Kabir Torsha, Gino Lim, Hadis Moazami Goudarzi, Radhe Mohan, David Grosshans, Wenhua Cao

**Affiliations:** ^1^ Department of Industrial and Systems Engineering University of Houston Houston Texas USA; ^2^ Department of Radiation Physics The University of Texas MD Anderson Cancer Center Houston Texas USA; ^3^ Department of Radiation Oncology The University of Texas MD Anderson Cancer Center Houston Texas USA

**Keywords:** IMPT, LET optimization, robust optimization

## Abstract

**Purpose:**

To perform a quantitative analysis of the trade‐off between robustness‐based and linear energy transfer (LET)‐based optimization of intensity‐modulated proton therapy for anatomically challenging pediatric brain tumor cases.

**Methods:**

Three pediatric brain tumor patients were included in this study. Three plans were generated for each case based on: (1) nominal optimization, without considering uncertainties and LET; (2) robust optimization, including proton range and patient setup uncertainties; and (3) LET optimization, considering increasing LET in the target and reducing LET in normal tissues. All plans were optimized with individually fine‐tuned objective weighing to obtain highest achievable target coverage and meet dose limits of critical structures considering robustness or LET criteria. We then evaluated the impact of robust optimization on LET distribution and assessed the robustness of the LET optimization plan, intercompared with the nominal plan. We also compared the dose and LET distributions, as well as biological effect, for each individual beam across the three plans.

**Results:**

Robust optimization consistently achieved robust target coverage among all cases, but it reduced the mean LET in the target by 8%–15% compared to nominal optimization and by 11%–20% compared to LET optimization. LET optimization was effective in reducing high LET in normal tissues. In patient cases, it reduced the maximum LET in the brainstem and spinal cord by 29%–41% and 13%–36%, respectively, compared to nominal optimization. Robust optimization also reduced high LET in the brainstem and spinal cord, but to a slightly lesser extent than LET optimization. Moreover, both robust and LET optimization resulted in less variation in the mean and maximum LETd for OARs compared to nominal optimization.

**Conclusion:**

An inherent conflict between robust target coverage and high LET in the target in IMPT planning was demonstrated in our study for pediatric brain tumor patients. Robust optimization resulted in lower LET not only in the target but also in nearby critical structures, such as brainstem and spinal cord. In contrast, LET optimization improved the LET distribution but worsened the plan robustness compared to nominal optimization. The trade‐off effect between LET enhancement and plan robustness can vary in respect to anatomical relationships and needs to be carefully evaluated in IMPT planning.

## 1 INTRODUCTION

Proton therapy shows a clinical advantage over conventional photon therapy due to its unique characteristics in spatial dose distribution.^1^ Proton beams deposit most of the dose over a confined distance near the end of the beam range, called Bragg peak. Intensity modulated proton therapy (IMPT), an advanced form of proton therapy, utilizes this Bragg peak to modulate beamlets in three dimensions to deliver highly conformal doses to target volumes with complex shapes.^2^, ^3^ Proton beams also show higher ionization density, which makes them more biologically effective at killing cells than conventional photon and electron radiation.^4^, ^5^ This biological effectiveness of protons can be addressed using relative biological effectiveness (RBE). RBE is the ratio of doses needed for a test radiation modality, such as proton therapy, to produce the same level of biological effect as a reference radiation source, that is, photons. A constant RBE value of 1.1 (meaning the protons are 10% more effective than photons) is used in current clinical practice, irrespective of beam quality, dose, tissue type, or tumor type, as recommended by the International Commission on Radiation Units and Measurements.^6^, ^7^ However, in vivo and in vitro studies have shown that proton RBE is not spatially constant, rather, varies as a function of physical and biological parameters,^8^, ^9^, ^10^, ^11^, ^12^, ^13^ such as linear energy transfer (LET), physical dose fractionation,^14^ linear‐quadratic model parameters, (*α*/*β*)_x_,^15^ tissue type,^16^ tissue structure,^17^ as well as clinical end point.

LET describes the average energy a charged particle deposits in tissue per unit distance traveled.^18^ As proton beams reach the end of their range, LET rises, and its value is particularly high around the distal edge of the Bragg peak, where proton RBE may exceed the clinically assumed value of 1.1.^19^ Several reports suggest that higher LET correlates with higher risk of damage to sensitive structures like the brainstem, brain, and lung during proton therapy, implying higher RBE than expected.^20^, ^21^, ^22^ To mitigate the risks associated with high‐LET regions, LET‐based evaluation and optimization techniques are increasingly adopted in proton therapy clinical practice.^19^

As an alternative to RBE‐based optimization, where consensus of RBE model is still lacking, extensive research on LET‐based optimization has been proposed in recent literature.^19^, ^23^, ^24^, ^25^, ^26^ Such methods use LET as a physical surrogate to guide doses in possibly high RBE away from OARs and concentrate them within the tumor. Since LET can be measured and modeled readily,^24^, ^27^ it offers a practical advantage over currently controversial biological models for protons.

Faught et al.^28^ studied the effects on dose averaged LET (LETd) across various IMPT treatments for four cases of pediatric brain cancer patients and an opposed lateral plan (phantom study). They included two types of uncertainty, that is, setup and range uncertainty, in their study to simulate clinical conditions. The proton beam range is significantly affected by tissue density variations,^29^ which leads to potential dose discrepancies from anatomic changes and uncertainties in converting computed tomography (CT) densities to stopping power.^30^ On the other hand, setup uncertainty that arises from patient positioning and equipment precision also affects dose delivery accuracy.^31^ In their study, Faught et al.^28^ used dose‐based planning without LETd‐based functions included in the optimization and found that the spatial distribution of LETd depended strongly on patient anatomy and treatment geometry. They also observed that increasing the range uncertainty in the optimization led to decreased LETd within the target in the phantom study. However, no consistent trend in mean or maximum LETd variations was observed in clinical cases.

Fredriksson et al.^32^ later examined the relationship between robust target coverage, dose uniformity, and LETd in carbon therapy, finding an inherent conflict between range uncertainty robustness and high LETd in larger targets for phantom cases. Rana et al.^33^ conducted a quantitative robustness analysis of LETd and constant RBE‐weighted dose in proton lung cancer plans and found that setup and range uncertainties impact LETd distribution homogeneity and reduce robustness. Some studies have included LET‐dependent functions to control LETd in the tumor directly. For example, Liu et al.^3^ proposed a LET‐guided robust optimization for head and neck cancer, achieving LETd reductions in OARs and a LETd boost within targets while maintaining physical dose and robustness. An et al.^34^ also optimized IMPT by incorporating both dose‐ and LET‐based objectives in robust optimization, and their study yielded similar results.

However, to date, the trade‐off between robust target coverage and optimal LET or biological effect has not been well evaluated and addressed in the literature. Understanding this trade‐off is crucial for practitioners to weigh the benefit of biological impact against plan robustness in treatment planning. Thorough assessment of differences in dose, LET, biological effect, and robustness among current clinical planning strategies, for example, robust optimization and LET optimization, is needed for reexamining current practice and development of new methods that can optimize the impact of this trade‐off. Note that LET optimization can also be implemented in robust optimization, but the notion of LET optimization in the present study does not include a robustness component. Our objective is to study both the best LET distribution without robustness criteria and the most robust plan with LET goals in IMPT planning, seen as two Pareto solutions in multicriteria optimization, and to assess their trade‐off.

In this study, we evaluate three optimization strategies in IMPT planning for pediatric brain tumor patients, focusing on both physical metrics such as physical dose and LETd, and biological metrics using biological effect and variable RBE weighted doses. We provide quantitative comparisons that clarify the magnitude of trade‐offs between these approaches. This analysis enables clinicians to objectively weigh the implications of each optimization method using interpretable, model‐driven metrics. The objectives of our study are four‐fold. First, we study the conflict between achieving target coverage and maximizing LETd in the tumor for IMPT treatment planning. To do this, we use three optimization approaches: nominal optimization, where we optimize the constant RBE‐weighted dose for the nominal scenario without uncertainties; robust optimization, which aims to optimize the constant RBE‐weighted dose across various uncertainty scenarios; and LET optimization, which considers both LETd and constant RBE‐weighted dose for optimizing in a nominal scenario without uncertainties. These three approaches directly assess the conflict between robust target coverage and high target LETd. Second, we compare the robustness of these three optimization approaches while maintaining clinically acceptable dosimetric quality. Third, we evaluate the effect of robust optimization in LETd distribution under different uncertainty scenarios. Finally, we compare the biological effect considering variable RBE for all three plans to evaluate the potential biological impact of the resulting LETd distributions for both target volumes and OARs.

## 2 MATERIALS AND METHODS

### 2.1 Treatment planning data

**FIGURE 1:**
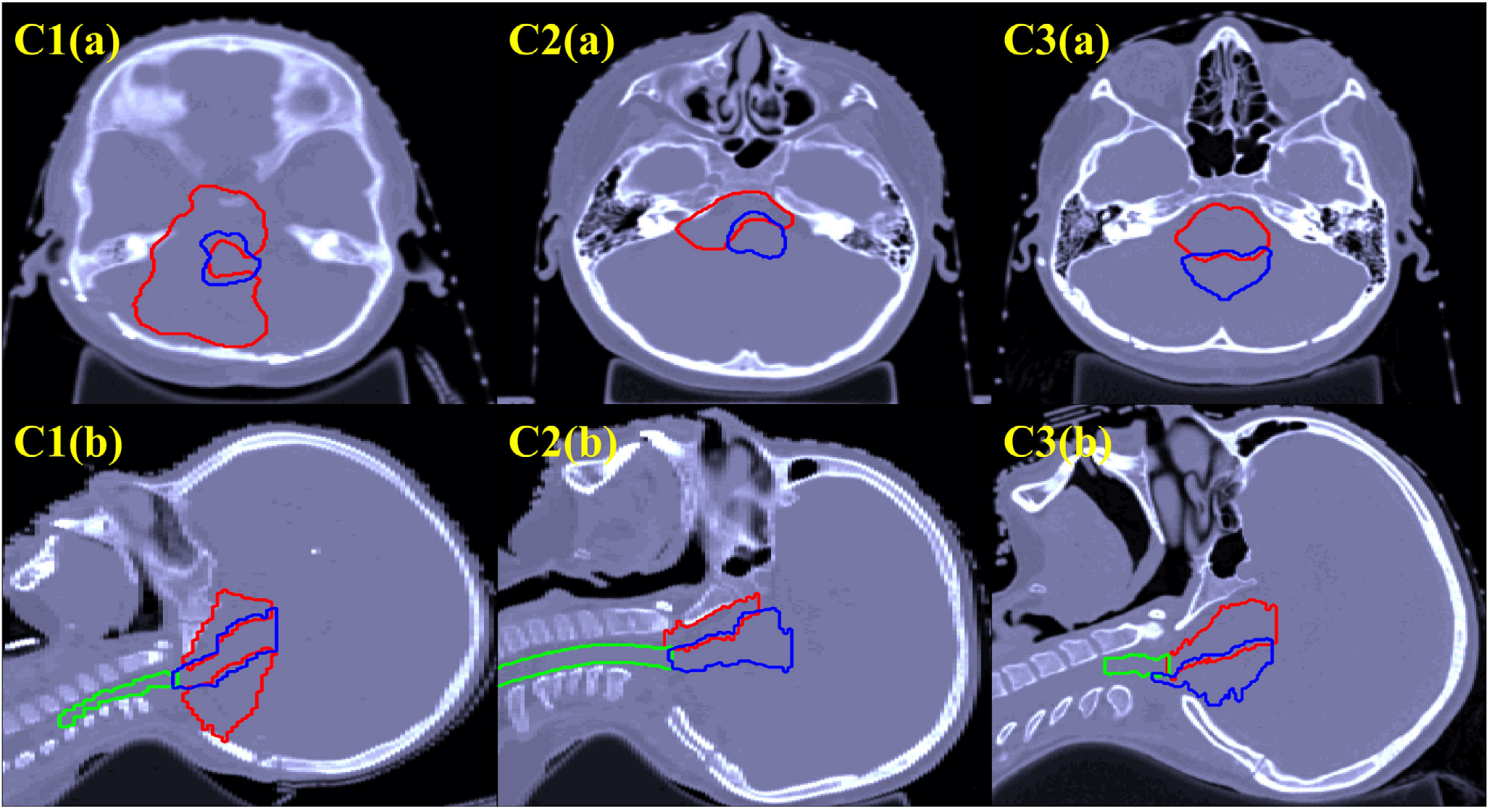
CT images of three patient cases (1–3), with axial views on the top row (a) and sagittal views on the bottom row (b). The bright red contour delineates the clinical target volume (CTV), the blue contour outlines the brainstem, and the green contour represents the spinal cord.

The three treatment‐planning strategies used in the current study are evaluated for three clinical cases of pediatric ependymoma from patients with complex‐shaped tumors located next to critical organs—the brainstem and spinal cord. Axial and sagittal computed tomography images for the patients are shown in Figure 1. For all three patients, a dose prescription of 54 Gy (constant 1.1‐RBE–weighted) is used, delivered in 30 fractions to the clinical target volume (CTV), with maximum voxel dose constraints set at 57 Gy (RBE) for the spinal cord and 60 Gy (RBE) for the brainstem.^35^ In this study, we use CTV only for prescription, without planning target volume (PTV) as used in a common practice in proton therapy for pediatric patients.^36^, ^37^ Details of the beam angles and beamlet numbers for each patient are provided in Table 1.

**TABLE 1:** Patient information and treatment planning parameters.

Case #	Beam angles (gantry, couch)	Number of beams	CTV size (cc)	Total number of voxels (CTV, brainstem, spinal cord)
1	(180,0), (270,15), (90,345)	3	55.27	2047 (1529, 391, 127)
2	(245,0), (180,0), (112,0), (315,0)	4	56.67	2099 (907, 719, 473)
3	(105,0), (255,0), (285,90)	3	40.88	1514 (680, 739, 95)

### 2.2 Optimization strategies

In this study, we implement and compare three IMPT planning strategies: nominal, robust, and LET optimization. Nominal optimization optimizes the constant RBE‐weighted dose for the nominal scenario without accounting for uncertainties. Robust optimization optimizes the constant RBE‐weighted dose across multiple scenarios to account for uncertainties. Lastly, LET optimization optimizes the LETd and constant RBE‐weighted dose for the nominal scenario without accounting for uncertainties.

For nominal optimization, we utilize the optimization routine implemented in matRad,^38^ which minimizes a weighted sum of objectives specified for dose limits. In the objective function, penalty weights are assigned for both underdosing and overdosing the target, as well as for overdosing the OARs. A constant RBE of 1.1 is applied, as commonly used in current clinical practice.^6^, ^7^

For robust optimization under uncertainties, several approaches are available, for example, objective‐wise worst‐case, voxel‐wise worst‐case, and composite worst‐case methods.^39^ In this study, we use the objective‐wise worst‐case approach in matRad,^38^ where the same objective function from nominal optimization is calculated and minimized for each of the uncertainty scenarios, that is, assuming all voxel doses are affected by one error realization.^39^

For LET optimization, we calculate the LET delivered to each voxel i by beamlet j at unit intensity, denoted as $L_{ij}$. LETd for voxel i, $L_{i}$, is defined over all pencil beam contributions as follows^4^:

(1) $$1\begin{array}{c} L_{i}=\frac{\sum_{j}D_{ij}L_{ij}w_{j}}{\sum_{j}D_{ij}w_{j}}. \end{array}$$

Here, $D_{ij}$ is the dose contribution from beamlet j to voxel i in unit intensity, $w_{j}$ is the intensity of beamlet j, and $L_{ij}$ is LET contribution from beamlet j to voxel i in unit intensity. The objective function for LET optimization in Equation (2) extends the standard dose‐based formulation by incorporating additional LETd‐related terms, enabling simultaneous optimization of both dose and LETd. The constraints remain the same as those used in nominal optimization. By including LETd terms in the objective, the optimization redistributes beamlet intensities to increase LET within the target and reduce LET in surrounding OARs, while maintaining acceptable target coverage and OAR sparing based on dose criteria.

(2) $$2F_{L}\left(w\right)=F_{D}\left(w\right)-\frac{\phi_{T}}{\left|N_{T}\right|}\sum_{i=1}^{N_{T}}L_{i}^{2}+\frac{\phi_{O}}{\left|N_{O}\right|}\sum_{i=1}^{N_{O}}L_{i}^{2}.$$

Here, $F_{D}\left(w\right)$ represents the conventional objective that penalizes deviations from prescribed dose levels in the target and OARs. Penalty weights $\phi_{T}$ and $\phi_{O}$ control the priorities of LETd in the target and OARs, respectively.

In this study, different sets of penalty weights are employed to guide the optimization process. For both nominal and robust optimization, weights in the objective function balance the trade‐off between maximizing target coverage and minimizing dose to OARs. In LET optimization, weights of LET objectives are used to control the relative priorities of LETd in the target and OARs. These penalty weights are fine‐tuned independently to achieve optimal treatment plans for each patient. We prioritize meeting OAR dose limits over target coverage according to our clinical practice for these cases. For OARs, we set clinical acceptability thresholds of 60 Gy(RBE) for the brainstem and 57 Gy(RBE) for the spinal cord. For CTV, its coverage, as evaluated by $D_{95\%}$, is optimized to be no less than 51 Gy(RBE). Plans are considered acceptable if these criteria are satisfied.

### 2.3 Scenario generation for robust optimization

In this study, we generate a total of nine scenarios per patient by considering both setup and range uncertainties. Six of these scenarios involve ±2.25 mm setup shifts applied independently along the anterior–posterior, left–right, and superior–inferior directions; two scenarios include ±3.5% range uncertainty; and one represents the nominal (unperturbed) case. For robust optimization, all nine scenarios are incorporated into the objective function to ensure robustness against uncertainties. On the other hand, nominal and LET optimization are performed using only the nominal scenario. Following optimization, dose and LETd distributions are recalculated across all nine scenarios.

### 2.4 Biological effect

We assess biological effect using two methods: variable RBE‐weighted dose, and product of LET and physical dose.^4^, ^23^, ^31^, ^34^, ^35^ The variable RBE‐weighted dose accounts for spatial variations in biological response by incorporating tissue‐specific parameters and LETd dependence along with physical dose, providing a more nuanced estimate than the constant RBE assumption. We use the RBE model introduced by McNamara et al.,^15^ with tissue‐specific parameters ${\left(\alpha /\beta \right)}_{x}=2$ for OARs and ${\left(\alpha /\beta \right)}_{x}=10$ for tumor.

We further assess the product of LET and physical dose as a surrogate for biological effect of, which is commonly adopted in the literature.^4^, ^23^, ^31^, ^34^ Specifically, if the RBE‐weighted dose is approximated by the form: D+cLETd×D, where D is the physical dose and c is a scaling constant (e.g., set to 0.04μm/keV^31^), the term, (cLETd×D) represents the LET‐dependent biological dose component in this simplified linear RBE model. We compute these two metrics from all plans for comparative analysis.

### 2.5 Computation environment

We use matRad,^38^ an open‐source treatment planning system for radiotherapy in MATLAB, to create all treatment plans and generate dose and LET influence matrices. The voxel size is set to 3 × 3 × 3 mm^3^, with lateral spot spacing of 5 mm. For dose^40^ and LET^27^ calculation, we utilize analytical models provided by matRad. The beam model for dose and LET calculations is based on a generic proton base data set, called “*Generic*”^41^, included in matRad. It represents a generic beam line using a single Gaussian lateral spread model. As all IMPT optimization problems in this study are highly non‐convex, we use the Interior Point Optimizer (IPOPT),^42^ a solver for large‐scale nonlinear optimization problems included in matRad. All computations are performed on a laptop with an Intel Core i7 CPU (2.50 GHz) and 32 GB of RAM.

### 2.6 Evaluation methods

To evaluate the impact of different optimization strategies, we analyze various dosimetric measures, including dose‐volume histograms (DVHs), LET‐volume histograms (LEV‐VHs) for all scenarios, dose and LETd distributions, mean dose and LETd for the target, and maximum dose and LETd for the OARs. Each plan is normalized to achieve consistent $D_{95}$, defined as the dose received by 95% of the target volume. Additionally, when comparing LETd distributions and mean and maximum LETd, we set the LETd value to zero in organ regions where the dose is 1 Gy or less. This threshold ensures that LETd values are calculated only for regions where the dose is clinically relevant, preventing the amplification of low‐dose areas that are unlikely to contribute meaningfully to treatment outcomes. For robustness analysis, we use the worst‐case minimum and worst‐case maximum across all uncertainty scenarios and the bandwidth between these extremes. Finally, for biological effect analysis, we compute the mean biological effect ($BE_{mean}$), for example, LET‐weighted dose for the target and the maximum biological effect ($BE_{max}$) for the OARs for the nominal scenarios across all three optimization strategies.

## 3 RESULTS

### 3.1 Conflict between target coverage and LET distribution

**FIGURE 2:**
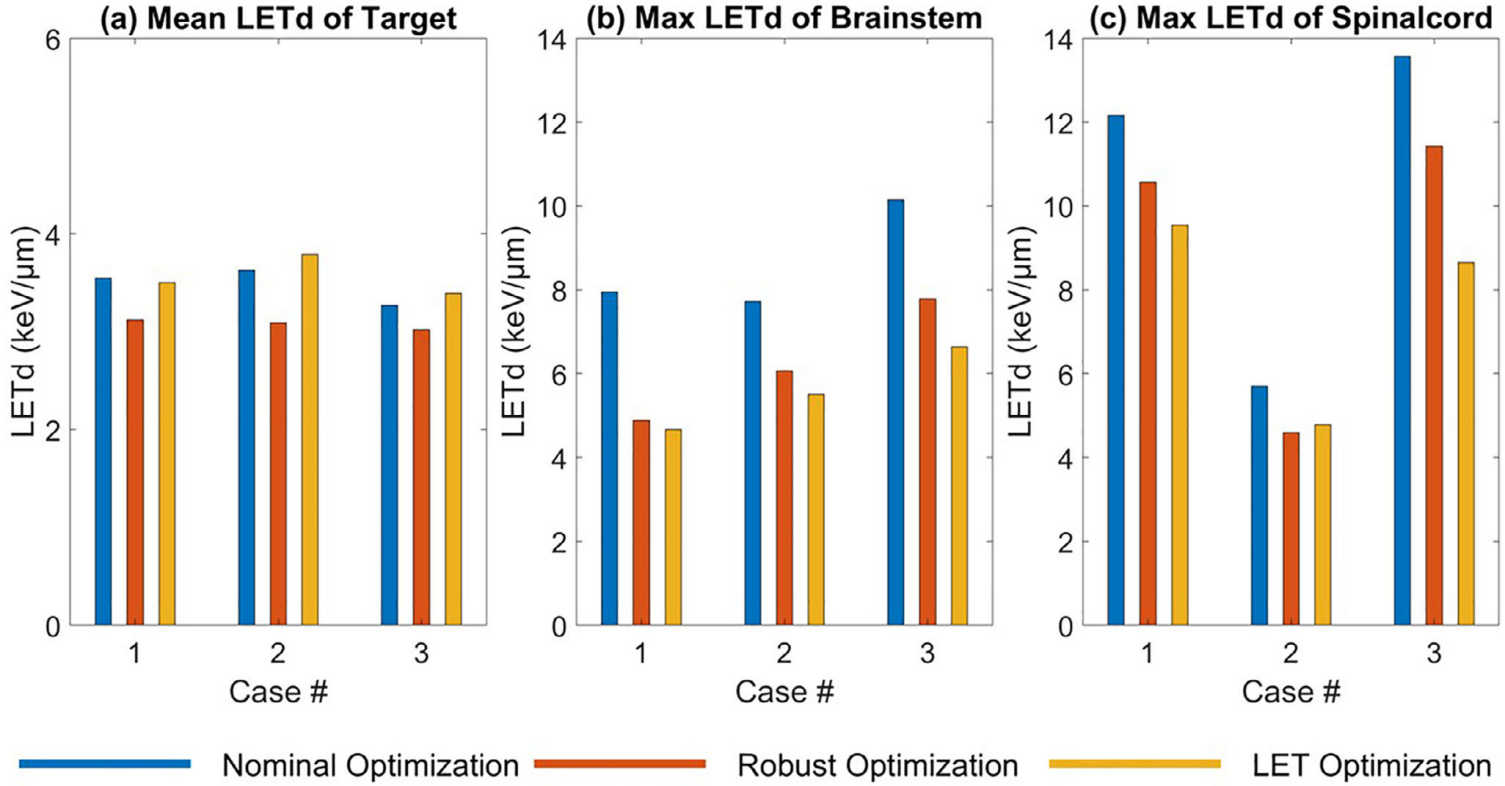
Nominal mean LETd for the target (a), maximum LETd for the brainstem (b), and maximum LETd for the spinal cord (c) from nominal, robust, and LET optimization.

Figure 2 shows the mean LETd within the target and the maximum LETd values in OARs under the nominal scenario without uncertainty across three optimization plans. These results indicate that incorporating the LET objectives increases the mean LETd within the target (Figure 2a) and effectively reduces the maximum LETd in the brainstem and spinal cord (Figure 2b,c), compared to nominal and robust optimization plans that do not include LET objectives, except for the maximum LETd in spinal cord for Case 2. For maximum LETd in the spinal cord of Case 2, LET optimization yields a slight increase relative to robust optimization. Additionally, Figure 2a shows that the robust optimization plan consistently shows a reduced mean LETd in the target, with decreases of 11% for Cases 2 and 3 and 18% for Case 1 relative to LET optimization. Although LET optimization generally achieves the lowest maximum LETd (except for the spinal cord in Case 2), robust optimization still lowers maximum LETd in OARs compared to nominal optimization. For the brainstem, the maximum LETd in robust optimization is 39%, 21%, and 23% lower than nominal optimization for Cases 1, 2, and 3, respectively. For the spinal cord, these reductions are 13%, 19%, and 16% for Cases 1, 2, and 3, respectively. Detailed values for dose and LETd for all plans are shown in Appendix, Tables A1–A6.

**FIGURE 3:**
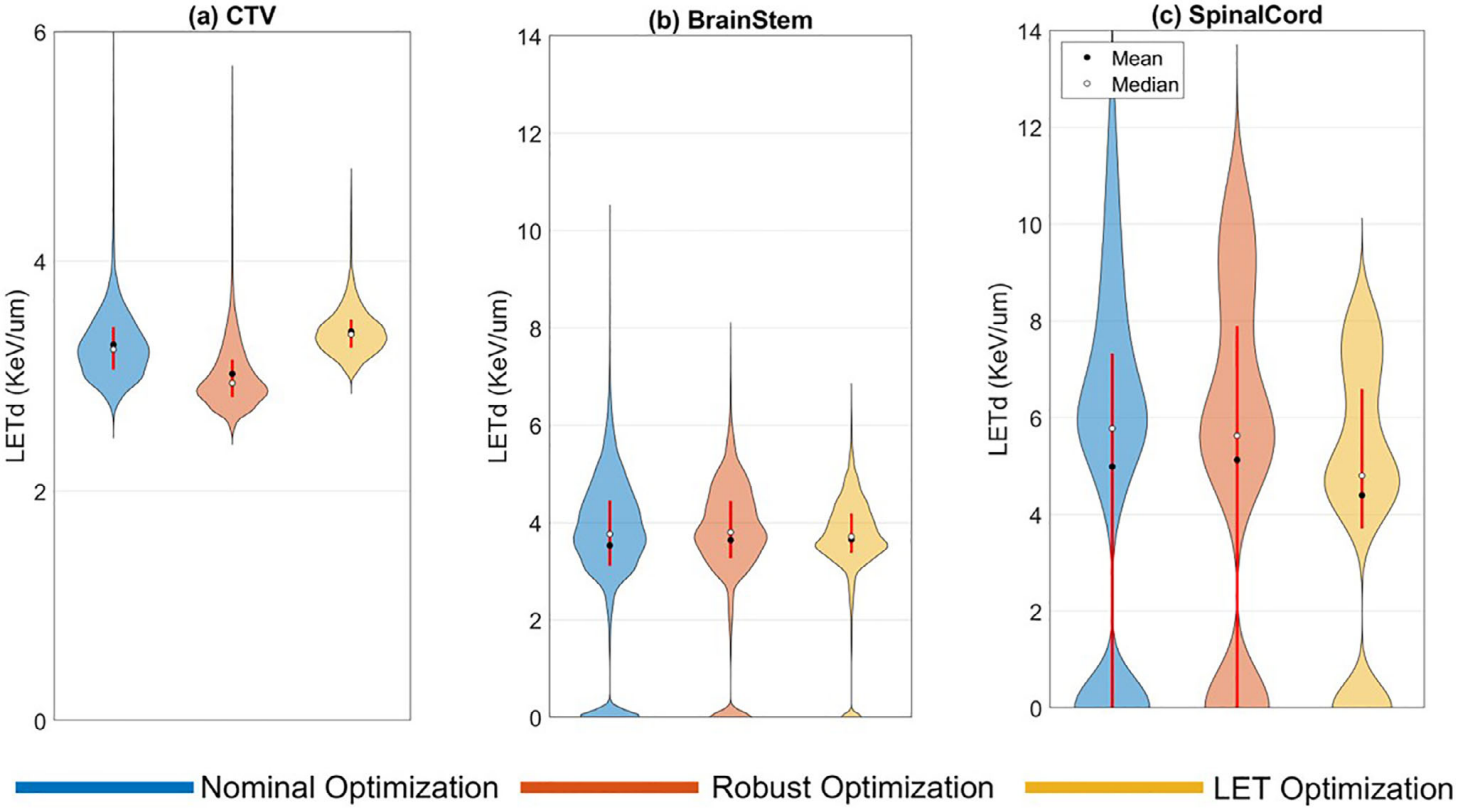
Violin plots showing the LETd distribution in the target (a), brainstem (b), and spinal cord (c) for Case 3.

For further evaluation of the optimization plannings, Figure 3 depicts a spatial distribution of LETd values within the target and OARs for Case 3. The violin plots show a detailed view of LETd distribution, uniformity, and variability across the three optimization plans. The width of each violin plot indicates the number of voxels that have a specific LETd level along the Y‐axis. Figure 3a shows that the median LETd value, located at the widest part of the LET optimization violin plot (in yellow), is higher than that of the other two optimization plans, indicating that more than half of the target voxels receive a relatively high LETd level in LET optimization compared to the other plans. Additionally, the shorter height of the LET optimization violin plot indicates a more uniform LETd distribution within the target. For robust optimization, Figure 3a shows the lowest median LETd within the target. This distribution suggests that robust optimization delivers a lower LETd level to a larger proportion of target cells compared to the LET and nominal optimization plans.

Figure 3b,c illustrate the LETd distribution in the brainstem and spinal cord, respectively. For both OARs, the shortest height of the violin plots for LET optimization indicates the lowest maximum value and highest uniformity among the three plans. Furthermore, the small gap between the mean and median values in LET optimization indicates a more symmetrical LETd distribution. Robust optimization shows similar characteristics in a less pronounced manner. In contrast, nominal optimization consistently shows the widest LETd range in all organs, indicating a higher maximum LETd value and greater variability.

**FIGURE 4:**
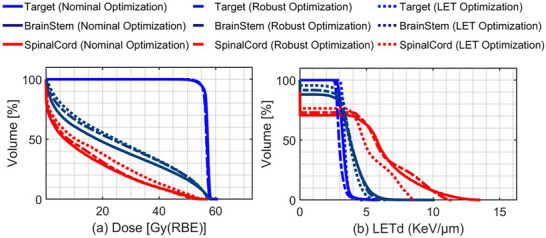
Left: dose‐volume histogram for Case 3 across all three optimization plans. Right: LETd‐volume histogram for Case 3 across all three optimization plans.

Figure 4 shows the DVH and LETd‐volume histogram (LETd‐VH) for Case 3 in the nominal scenario. The DVH shows that all three optimization plans (nominal, robust, and LET) provide comparable target coverage, as evidenced by the overlapping target lines. However, the LETd‐volume histogram shows the differences in LETd distribution for the target, with robust optimization showing the lowest LETd values compared to the other two plans.

**FIGURE 5:**
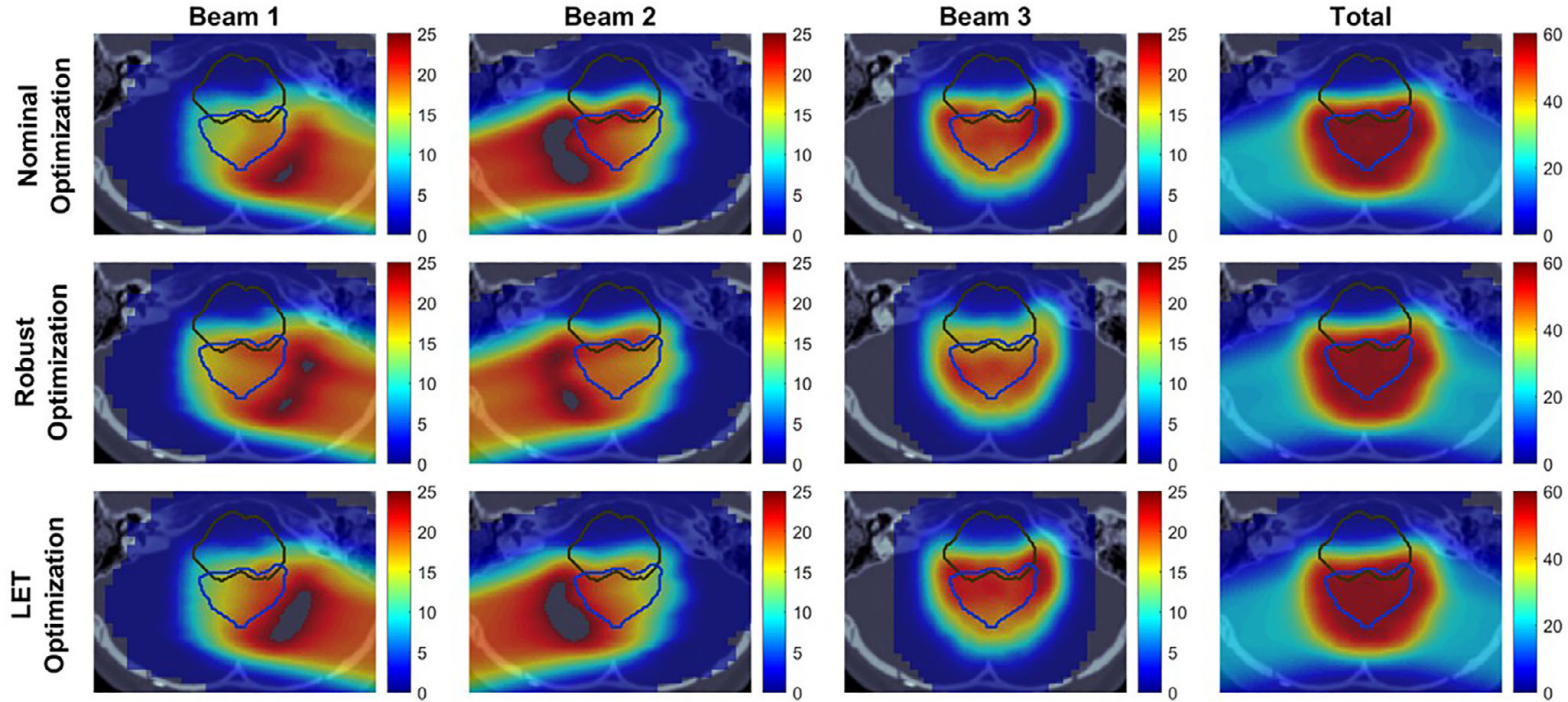
Axial view of constant RBE‐weighted dose distribution in Case 3. The bright blue contour represents the target region, and the dark blue contour outlines the brainstem. The black contour along the beam path indicates regions with higher doses that exceed the displayed scale range.

**FIGURE 6:**
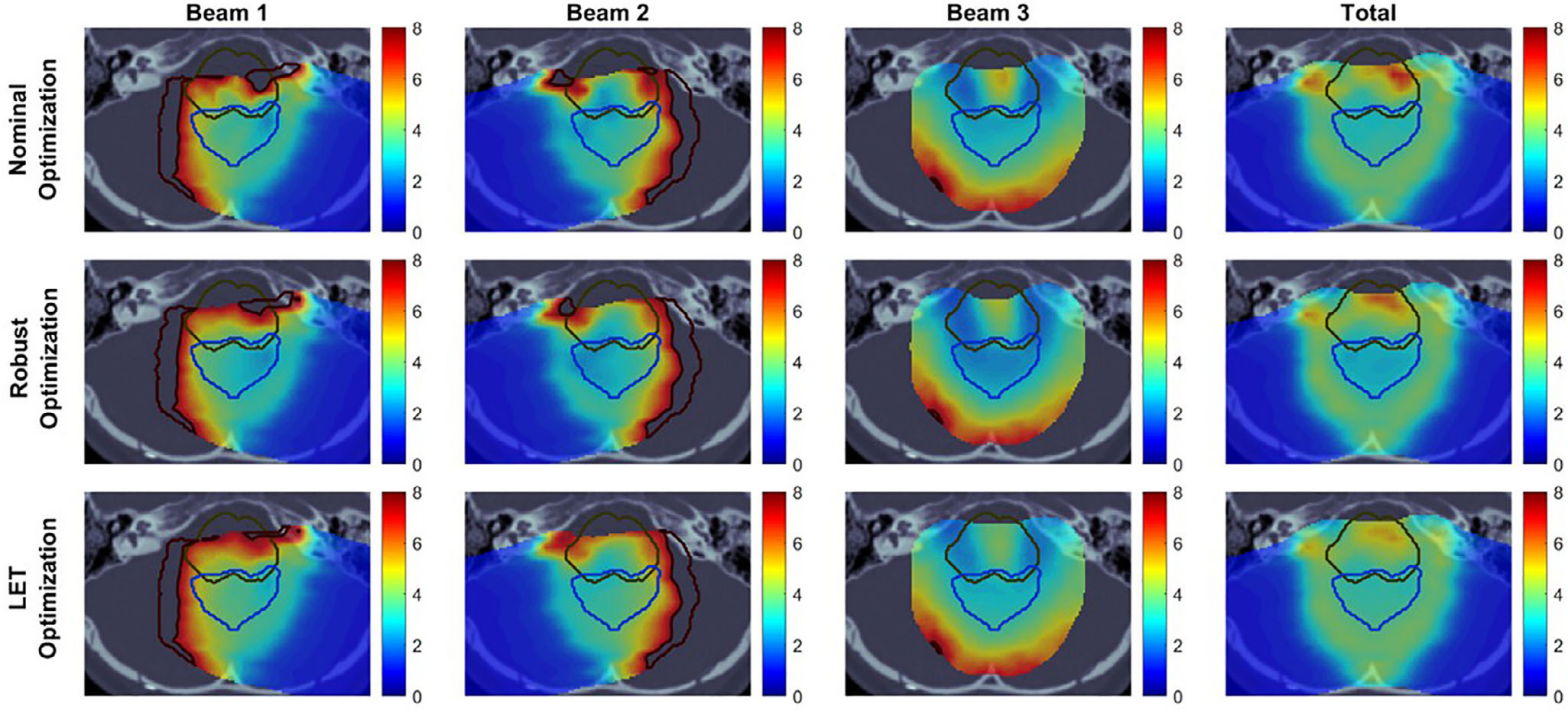
Axial view of LETd distribution in Case 3. The bright blue contour represents the target region, and the dark blue contour outlines the brainstem. The dark red contour along the beam path indicates regions with higher LETd values that exceed the displayed scale range.

To further explain the LET distribution with dose distribution, Figures 5 and 6 display the per‐beam and total dose distributions alongside the corresponding LETd distributions in an axial view for Case 3. Figure 5 illustrates the RBE‐weighted dose distribution, where the bright blue contour outlines the target region, and the dark blue contour represents the brainstem. The black contour along the beam path for beams 1 and 2 indicates regions with dose levels exceeding the scale range. In the nominal and LET optimization plans, beams 1 and 2 show concentrated high‐dose areas extending the scale near the target region. Compared to that, the robust optimization plan presents a more uniform dose distribution along the beam paths, which leads to moderate changes in the total dose in the target if the density changes due to uncertainty. In contrast, the concentrated high‐dose regions in the nominal and LET optimization plans may lead to over‐ or under‐dosing near the target under uncertainties, potentially compromising dose coverage under uncertain conditions.

Figure 6 shows the axial view of LETd distribution for the target and brainstem regions. The dark red contour indicates areas with LETd values exceeding the scale. The increased dose concentrations along the directions of beams 1 and 2 in LET optimization (Figure 5) lead to higher LETd within the target (Figure 6). In contrast, robust optimization is designed to manage uncertainties by distributing doses more evenly, which results in lower LETd within the target. The directions of beams 1 and 2 are visible in the axial view, while the direction of beam 3 is visible in the sagittal view. Figures A2 and A3 in Appendix illustrate the dose distribution and LETd distribution, respectively, in the sagittal view for Case 3. In Figure A2, nominal and LET optimization plans concentrate doses along the direction of beam 3 near the target, which results in higher LETd within the target. In contrast, robust optimization shows a more uniform dose distribution along the beam 3 path, which leads to a lower LETd in the target.

### 3.2 Biological effect

For the biological effect analysis of each optimization plan, variable RBE‐weighted doses are calculated using the RBE model introduced by McNamara et al.^15^ Table 2 presents the results for all plans and patients.

**TABLE 2:** Variable RBE‐weighted doses (Gy[RBE]) for the target (mean), brainstem (maximum), and spinal cord (maximum) across three pediatric brain tumor cases, comparing Nominal, Robust, and LET‐based optimization plans.

	Case 1			Case 2			Case 3		
	Nominal	Robust	LET	Nominal	Robust	LET	Nominal	Robust	LET
Target Mean	55.6	55.8	55.6	56.0	56.1	56.9	56.8	56.3	56.6
Brainstem Max	67.0	64.6	65.1	65.9	64.4	64.4	66.3	66.2	65.8
Spinal cord Max	46.6	52.7	54.0	63.5	62.3	57.6	67.8	65.8	65.7

For the target, the mean variable RBE‐weighted doses are comparable (within 2%) across optimization strategies. For the brainstem, robust and LET optimization results in the lower maximum dose (2% in average) than nominal optimization for all cases. For the spinal cord, the same trend is shown except for Case 1 (reduction in 2% to 9%), where increased maximum variable RBE‐weighted dose (over 10%) is seen in robust and LET optimization plans. This is mainly due to the spatial relationship between the spinal cord and the target for Case 1 and will be explained with more details in Discussion.

We further evaluate the cLETxD for all IMPT plans. Table 3 presents the mean cLETxD on the target and OARs for all optimization strategies. Robust optimization shows the lowest mean cLETxD in the target in all patient cases, while LET optimization shows consistently higher cLETxD, specifically by 11% in Case 1, 27% in Case 2, and 3% in Case 3, compared to robust optimization. For the brainstem, robust optimization shows a lower maximum cLETxD than LET optimization in all three cases. For the spinal cord, LET optimization results in higher (around 10%) maximum cLETxD in Cases 1 and 2 compared to robust optimization. In Case 3, LET optimization shows a lower maximum cLETxD (10%) than robust optimization.

**TABLE 3:** cLET×D for the target (mean), brainstem (maximum), and spinal cord (maximum) across three pediatric brain tumor cases, comparing Nominal, Robust, and LET‐based optimization plans.

	Case 1			Case 2			Case 3		
	Nominal	Robust	LET	Nominal	Robust	LET	Nominal	Robust	LET
Target Mean	7.2	6.4	7.1	7.4	6.3	8.1	6.8	6.2	7.0
Brainstem Max	10.9	8.0	8.9	8.7	7.0	8.9	9.1	8.0	8.1
Spinal cord Max	7.2	6.6	7.0	8.2	7.6	8.4	11.7	10.3	9.2

### 3.3 Plan robustness

**FIGURE 7:**
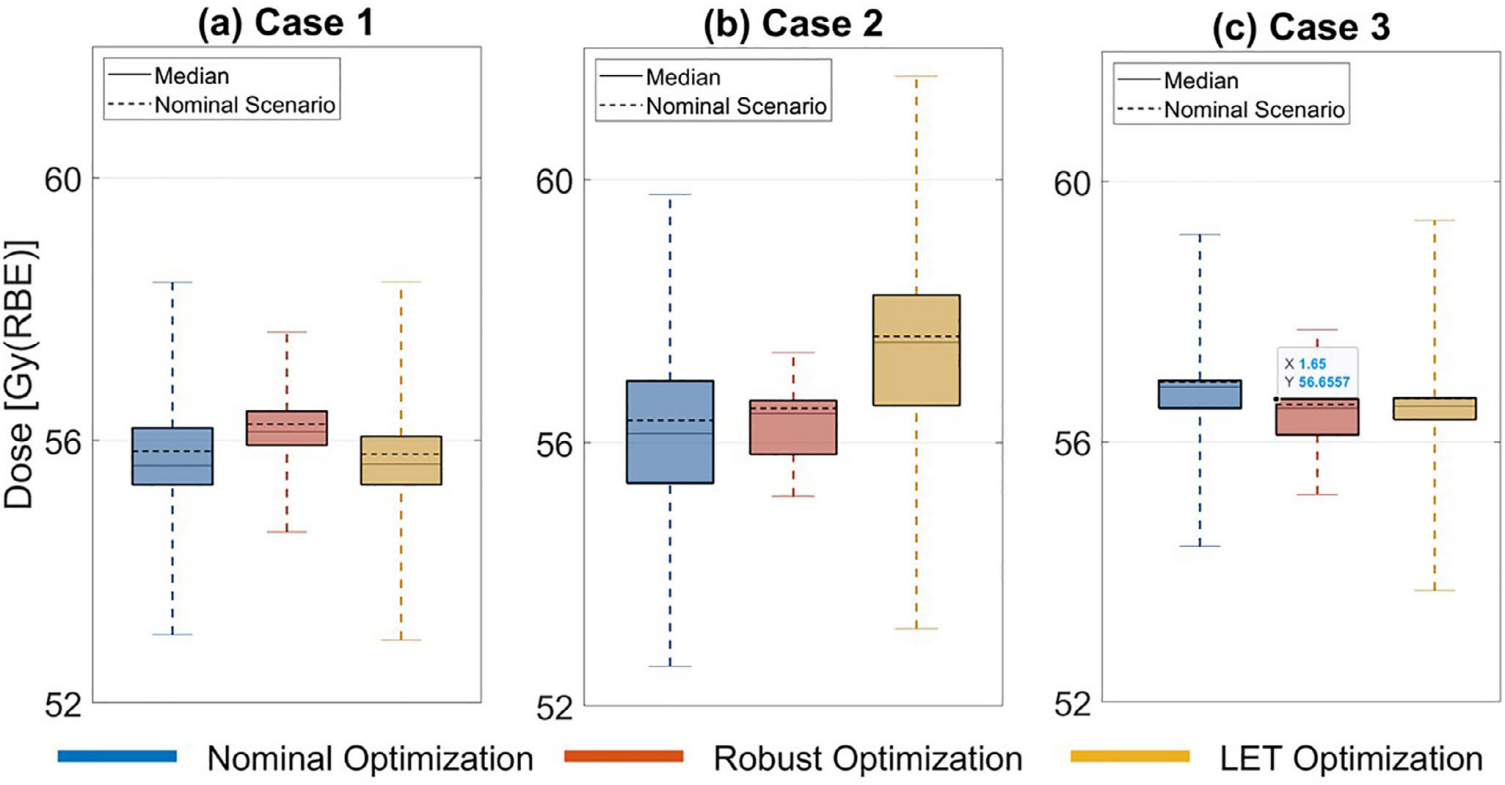
Boxplots comparing mean dose in the target for nominal, robust, and LET optimization plans in the nominal scenario and other scenarios under uncertainties for Case 1 (a), Case 2 (b), and Case 3 (c).

In the case of robustness analysis, Figure 7 shows that robust optimization consistently achieves the most consistent target coverage in terms of mean dose across all three pediatric cases. The consistency of a parameter is indicated by the mean bandwidth, which is the difference between the lowest and highest mean dose across all scenarios. In Case 1, the mean dose bandwidth in robust optimization is 43% narrower than nominal optimization and 44% narrower than LET optimization. For Case 2, the mean dose bandwidth is 70% and 74% narrower than nominal and LET optimization, respectively, and for Case 3, it is 47% and 56% narrower. A similar trend is observed in the bandwidths for brainstem and spinal cord maximum doses.

**FIGURE 8:**
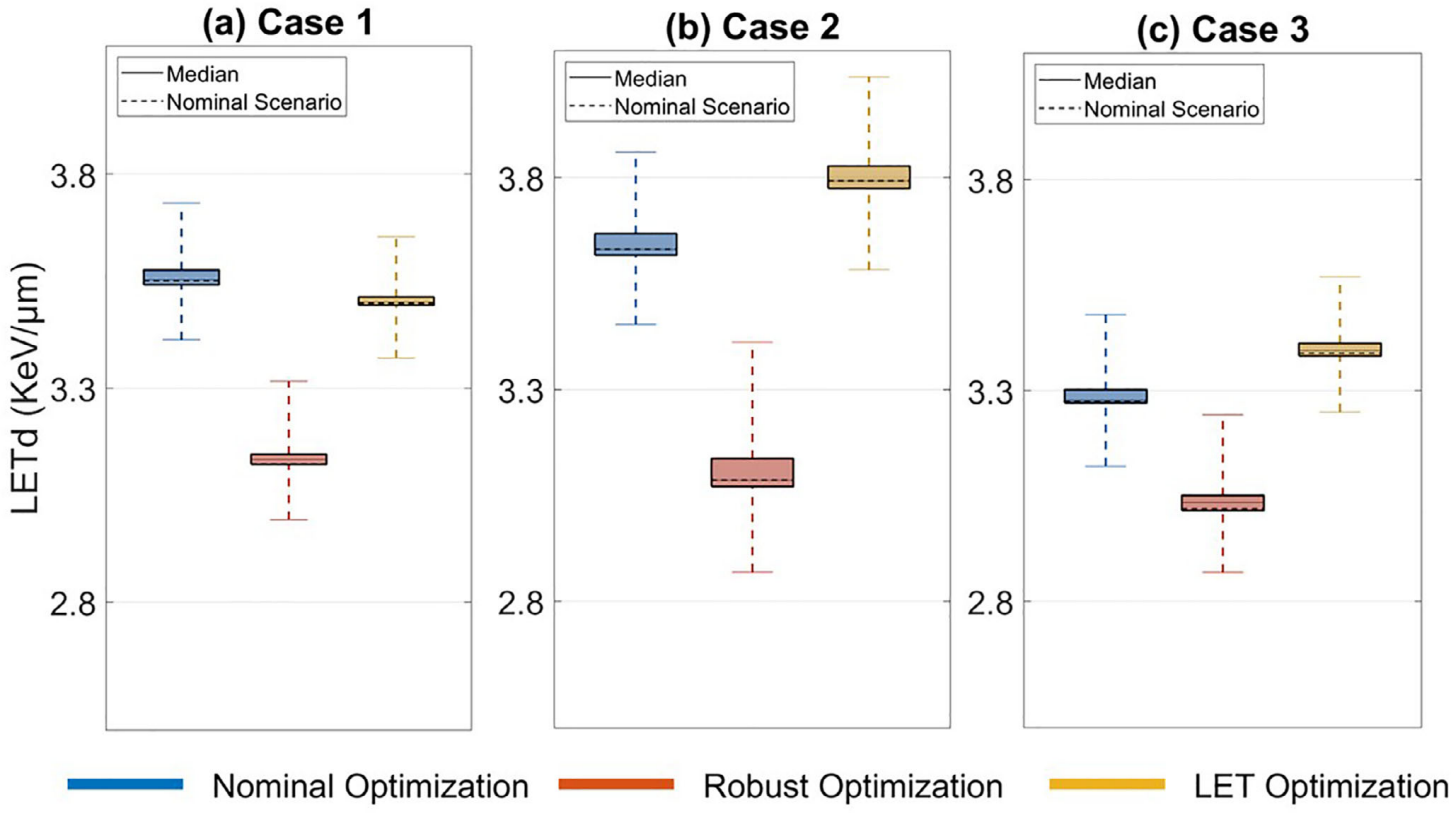
Boxplots comparing mean LETd in the target for nominal, robust, and LET optimization plans in the nominal scenarios and other scenarios under uncertainties for Case 1 (a), Case 2 (b), and Case 3 (c).

In terms of robustness in mean LETd in the target, Figure 8 does not show any consistent pattern across the three cases. Detailed robustness statistics for all three patients are listed in Tables A7‐A12 in Appendix.

## 4 DISCUSSION

In this study, we compared three IMPT treatment plans—nominal optimization, robust optimization, and LET optimization, and quantified the inherent conflict between achieving robust target coverage and maximizing LETd in the target. We evaluated three pediatric brain cancer cases to assess the applicability of our findings in clinical scenarios. Our results demonstrated that the trade‐off between robustness and high LETd persists in patient cases while maintaining dosimetrically acceptable dose levels in the target.

In contrast to previous studies by Faught et al.,^28^ which reported inconsistent trends in LETd variations in clinical pediatric brain tumor cases, our findings showed a consistent pattern across clinical cases. As shown in Figure 5 and Figure A2, robust optimization produced a flatter dose distribution with a “flatter” spread‐out Bragg peak along the beam paths (mainly in lateral oblique beams) compared to the nominal and LET optimization plans, resulting in more robust target coverage, as shown in Figure 7. Since most of the dose delivered in the central region of the target came from the plateau before the Bragg peak, where the particles have relatively high energy and low LET, the mean LET in the target was lower in the robust optimization plan than in the other two plans. In contrast, in LET optimization, we observed “ramping down” dose patterns along the beam paths, similar to those observed in nominal optimization. By adding LET objective in LET optimization, a steeper “ramping down” dose pattern was seen in individual beams compared to nominal optimization, which led to more particles with low energy and high LET in the target. Our outcome is consistent with findings by Fredriksson et al,^32^ who also reported a similar conflict between robust range uncertainty management and high LETd in the target manifested in carbon ion therapy.

In evaluating LET distribution in critical structures, the robustly optimized plans led to reduced maximum LET in the OARs compared to nominally optimized plans, consistently among patient cases (Figure 2). For example, robust optimization lowered maximum LETd by 21%–39% for the brainstem and 13%–17% for the spinal cord, compared to nominal plans of the three patient cases. In the meantime, LET optimization was able to reduce maximum LETd in the brainstem and spinal cord by 29%–41% and 13%–36%, respectively, compared to nominal plans. In other words, standard robust optimization was advantageous in limiting high LET in OARs, in a slightly smaller magnitude than LET optimization, even without using any LET objective in plan optimization.

For the spinal cord in Cases 1 and 3 under nominal optimization, the maximum LETd appeared higher than other plans, near 12 keV/µm. However, this occurred in a small fraction of the spinal cord volume—0.04% of voxels (one voxel) in Case 1 and 1.6% in Case 3. In Case 3, the maximum RBE‐weighted dose (in the simplistic linear RBE model) of these high‐LETd voxels was 4.66 Gy. Note that voxels receiving 1 Gy or less were excluded in LETd calculation in our study Some recent methods in the literature, instead, proposed to use relatively high threshold of physical dose (e.g. 40 Gy) in biological optimization or evaluation.^43^ Given the low dose and limited spatial extent, these high‐LETd voxels are of little clinical impact. Moreover, we did not observe such high maximum LETd in the spinal cord for robust optimization. As discussed earlier, in robust optimization, most of the dose delivered in the central part of the target came from the high‐energy‐low LET particles from the plateau before the Bragg peak, which led to lower mean LETd in the target, compared to the other optimization strategies. As there is no restriction of high‐LETd, in nominal optimization, such spillover cannot be avoided.

When evaluating variable RBE weighted doses among various plan (Table 2), robust optimization and LET optimization achieved very similar mean doses in target. The largest difference was seen in Case 2, where LET optimization yielded a mean dose 1.4% higher than robust optimization, indicating a slightly increase of tumor control probability. Robust and LET optimization also resulted in similar maximum OAR doses in all cases (difference within around 1 Gy), except that LET optimization achieved a maximum spinal cord dose 7.5% (4.7 Gy) lower than robust optimization in Case 2, indicating a clear advantage in reducing normal tissue complication probability for the spinal cord.

**FIGURE 9:**
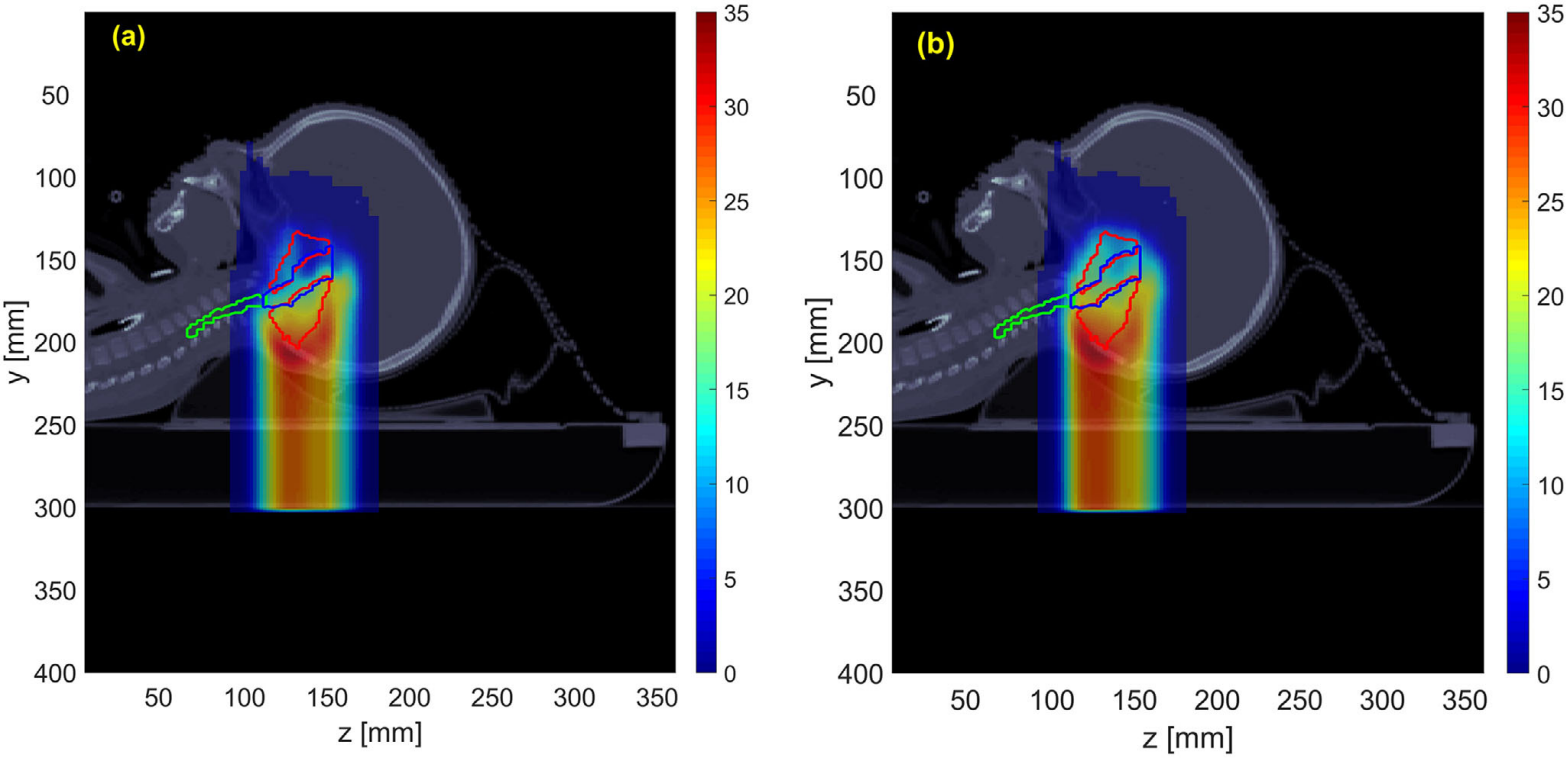
Physical dose distribution from Beam 1 for Case 1: (a) Nominal optimization and (b) LET optimization. The red contour delineates the clinical target volume (CTV), the blue contour outlines the brainstem, and the green contour represents the spinal cord. Compared to nominal optimization, LET optimization distributes more high‐dose region distally within the target, exposing higher dose to the spinal cord.

Interestingly, in Case 1, both robust and LET optimization increased the maximum variable RBE‐weighted dose to the spinal cord by over 13%, unlike other cases. This could be explained by its anatomical characteristics (Figure 1), where the C‐shaped target wraps around the brainstem with an overlapping region (22% of the brainstem voxels), and the spinal cord (superior‐inferior direction) is slightly more distant to the target (without target and spinal cord overlap), unlike the other 2 cases. Because of the beam arrangement and the spatial relationship between the target and the spinal cord, nominal optimization was able to spare the spinal cord more effectively than the other 2 plans. Robust optimization led to higher physical dose spill to the spinal cord for maintaining target coverage against range and setup uncertainties. LET optimization also increased dose to the spinal cord for the purpose of avoiding high LET in the brainstem. As an example, Figure 9 shows the physical dose distribution from the posterior beam (Beam 1) for nominal and LET optimization. The nominal plan only focuses on covering the posterior target and better spares the spinal cord compared to the LET plan. In contrast, LET optimization tends to cover both anterior and posterior targets with Beam 1 and avoid placing all Bragg peaks in front of the brainstem. As a result, the spinal cord is exposed with higher dose.

When evaluating the LET weighted dose in the simplistic linear RBE model (Table 3), cLETxD showed trends consistent with LETd in the target from different plans for all cases. Robust optimization resulted in the lowest mean cLETxD in the target among all plans. More importantly, robust optimization also resulted in lower cLETxD in the brainstem and the spinal cord than LET optimization for all cases, except the spinal cord for Case 3. This exception was attributed to the unique anatomical feature (Figure 1) and beam arrangement (Table 1) for this case. The target in Case 3 extends inferiorly beyond the brainstem to a large extent, unlike Case 1 and 2, and overlaps with the spinal cord. To maintain robust coverage to the target including the extended portion below the brainstem, robust optimization gave relatively higher weights to the two lateral oblique beams (see Beam 1 and 2 in Figure A1, Appendix), compared to LET optimization, subsequently led to high‐LET events in the spinal cord (Figure A2, Appendix). On the other hand, LET optimization exploited the vertex beam (see Beam 3 in Figure A1, Appendix) by weighting more towards Bragg peaks in proximal region of the beam and avoiding high LET in the spinal cord. Therefore, as shown in Figure A3 (Appendix), the final distribution of cLETxD by LET optimization better spared the spinal cord than robust optimization, which was not seen in other cases.

The present study is limited in that a combined robust LET optimization approach was not included. This aspect will be extended in our future work. However, separate analysis of robust and LET optimization methods essentially provides two non‐dominant solutions to the Pareto boundary of this multi‐objective optimization problem. As illustrated in this study and a previous one on heavy‐ion therapy,^3^ the conflict between target coverage robustness and ideal LET distribution is inherent in IMPT. Non‐conventional approaches, such as relaxing thresholds on target dose uniformity, or increasing the number of beams, even towards proton arc therapy potentially, may be effective in boosting LET in the target without degrading plan robustness and beneficial in certain clinical scenarios. Otherwise, as demonstrated in this study for anatomically challenging cases, benefits of LET optimization are limited. Robust optimization used in current clinical protocols not only generates robust IMPT plans but also limits increased biological effectiveness in close‐by critical structures even without LET optimization.

Besides robust optimization achieving the lowest variation in the target coverage among the three methods, a similar trend of reduced variation was also observed in the maximum doses for OARs. Our study also compared robustness in LETd in the three plans, showing that both robust and LET optimization resulted in reduced variation in mean and maximum LETd for OARs, compared to nominal optimization.

The current study is limited to a small number of patient cases, which constrains the generalizability of the results. In future work, we plan to extend our analysis to a broader range of patient populations and tumor geometries to assess the generalizability of our findings. We also acknowledge that minimizing planner bias was not a focus in this study. Instead, our priority was to ensure that each optimization method achieved the highest possible plan quality, as all three cases included in our study are anatomically challenging for planning and evaluation of biological effectiveness is of great importance. To that end, we individually fine‐tuned the planning strategies for each case to obtain the best possible dose and LET distributions under each optimization approach. A systematic evaluation of planner variability will be addressed in future investigations.

## 5 CONCLUSION

This study evaluated the conflict between robust target coverage and ideal LET distribution in IMPT planning based on three anatomically challenging pediatric brain cancer patients. By comparing nominal, robust, and LET‐based optimization approaches, our study showed that robust optimization led to lower LET in both target and nearby OARs, due to its emphasis on dose uniformity and robust coverage, compared to nominal optimization. In contrast, LET optimization was able to increase LET in the target and reduce high LET in OARs. However, when comparing robust and LET optimization in terms of biological effect integrating dose and LET, no clear advantage was found for one over the other, in either target coverage or OAR sparing.

These findings confirm that managing range and setup uncertainties through robust optimization limits its ability to elevate LET in the tumor, while LET optimization without robustness objectives cannot sustain plan robustness, highlighting an inherent trade‐off in IMPT planning. The magnitude of LET improvement if LET were optimized in a robust optimization framework has yet been tested. Overall, considering the tradeoff between plan robustness and potential biological effectiveness enhancement is important in IMPT treatment planning, and the optimal decision in selecting priorities could be made for individual patients under specific clinical scenarios.

## AUTHOR CONTRIBUTIONS

FKT, GL and WC developed the study. FKT implemented the methods and wrote the manuscript. HMG assisted in data preparation and analysis. RM and DG provided clinical inputs to the study. All authors contributed to this study and approved the submitted manuscript.

## CONFLICT OF INTEREST STATEMENT

The authors declare no conflicts of interest.

## Supporting information



Supporting Information
