# Supplementary material for: Quantitative analysis of robustness‐based versus LET‐based optimization in intensity‐modulated proton therapy for pediatric brain tumors
Source: J Appl Clin Med Phys. 2026 Feb 18;27(2):e70472. doi: 10.1002/acm2.70472 (PMC12914345; doi:10.1002/acm2.70472)
Supplement: Supplementary file 1 — Supporting Information [file ACM2-27-e70472-s001.docx]

Appendix A

**Table A1**. Mean dose [Gy(RBE)] in the target for different optimization plans.

| Case # | Nominal optimization | Robust optimization | LET optimization |
| --- | --- | --- | --- |
| 1 | 55.83 | 56.25 | 55.79 |
| 2 | 56.34 | 56.52 | 57.61 |
| 3 | 56.92 | 56.57 | 56.66 |

**Table A2**. Mean and maximum dose [Gy(RBE)] in the brainstem for different optimization plans.

| Case # | Nominal optimization | | Robust optimization | | LET optimization | |
| --- | --- | --- | --- | --- | --- | --- |
|  | **Mean** | **Max** | **Mean** | **Max** | **Mean** | **Max** |
| 1 | 40.60 | 57.58 | 43.74 | 57.98 | 45.10 | 57.77 |
| 2 | 24.93 | 58.17 | 26.47 | 58.11 | 27.01 | 59.74 |
| 3 | 23.17 | 58.25 | 25.71 | 58.68 | 26.60 | 57.91 |

**Table A3**. Mean and maximum dose [Gy(RBE)] in the spinal cord for different optimization strategies.

| Case # | Nominal optimization | | Robust Optimization | | LET optimization | |
| --- | --- | --- | --- | --- | --- | --- |
|  | **Mean** | **Max** | **Mean** | **Max** | **Mean** | **Max** |
| 1 | 3.04 | 36.17 | 4.88 | 41.52 | 5.66 | 44.76 |
| 2 | 2.81 | 55.31 | 3.95 | 55.46 | 3.85 | 56.18 |
| 3 | 13.58 | 57.28 | 14.19 | 55.55 | 16.69 | 57.31 |

**Table A4.** LETd (keV/$\mu$m) values in the target for different optimization plans.

| Case # | Nominal optimization | | Robust optimization | | LET optimization | |
| --- | --- | --- | --- | --- | --- | --- |
|  | **Mean** | **Max** | **Mean** | **Max** | **Mean** | **Max** |
| 1 | 3.55 | 5.95 | 3.12 | 4.61 | 3.50 | 4.99 |
| 2 | 3.63 | 4.55 | 3.09 | 4.27 | 3.79 | 4.63 |
| 3 | 3.27 | 5.97 | 3.02 | 5.61 | 3.39 | 4.74 |

**Table A5.** LETd (keV/$\mu$m) values in the brainstem for different optimization plans.

| Case # | Nominal optimization | | Robust optimization | | LET optimization | |
| --- | --- | --- | --- | --- | --- | --- |
|  | **Mean** | **Max** | **Mean** | **Max** | **Mean** | **Max** |
| 1 | 4.31 | 7.95 | 4.31 | 4.88 | 3.69 | 4.67 |
| 2 | 3.43 | 7.72 | 3.09 | 6.07 | 3.38 | 5.50 |
| 3 | 3.53 | 10.14 | 3.64 | 7.78 | 3.65 | 6.64 |

**Table A6.** LETd (keV/$\mu$m) values in the spinal cord for different optimization plans.

| Case # | Nominal optimization | | Robust optimization | | LET optimization | |
| --- | --- | --- | --- | --- | --- | --- |
|  | **Mean** | **Max** | **Mean** | **Max** | **Mean** | **Max** |
| 1 | 3.01 | 12.16 | 2.39 | 10.56 | 2.25 | 9.54 |
| 2 | 0.53 | 5.50 | 0.53 | 4.59 | 0.56 | 4.78 |
| 3 | 4.99 | 13.57 | 5.12 | 11.42 | 4.40 | 8.65 |


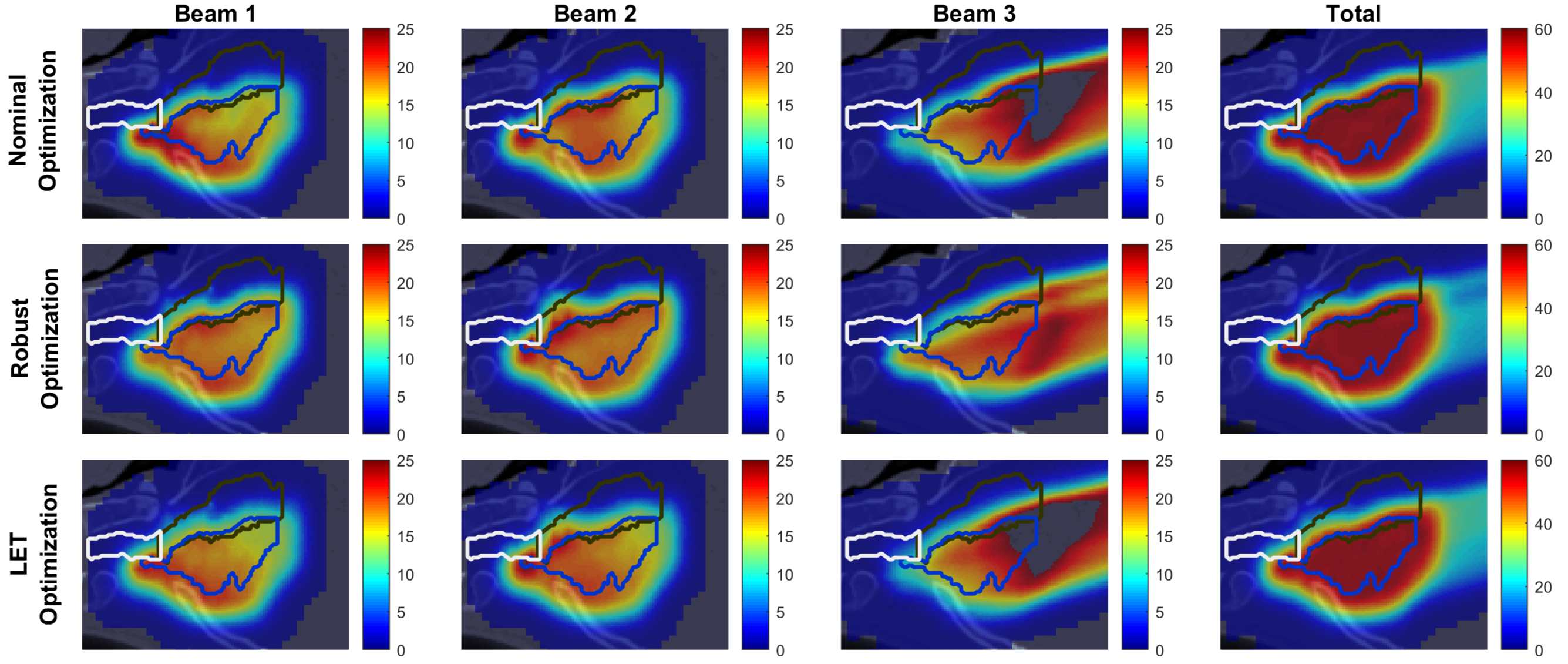


**Figure A1.** Sagittal view of constant RBE-weighted dose distribution in Case 3. The bright blue contour represents the target region, while the dark blue contour outlines the brainstem. The black contour along the beam path indicates regions with higher doses that


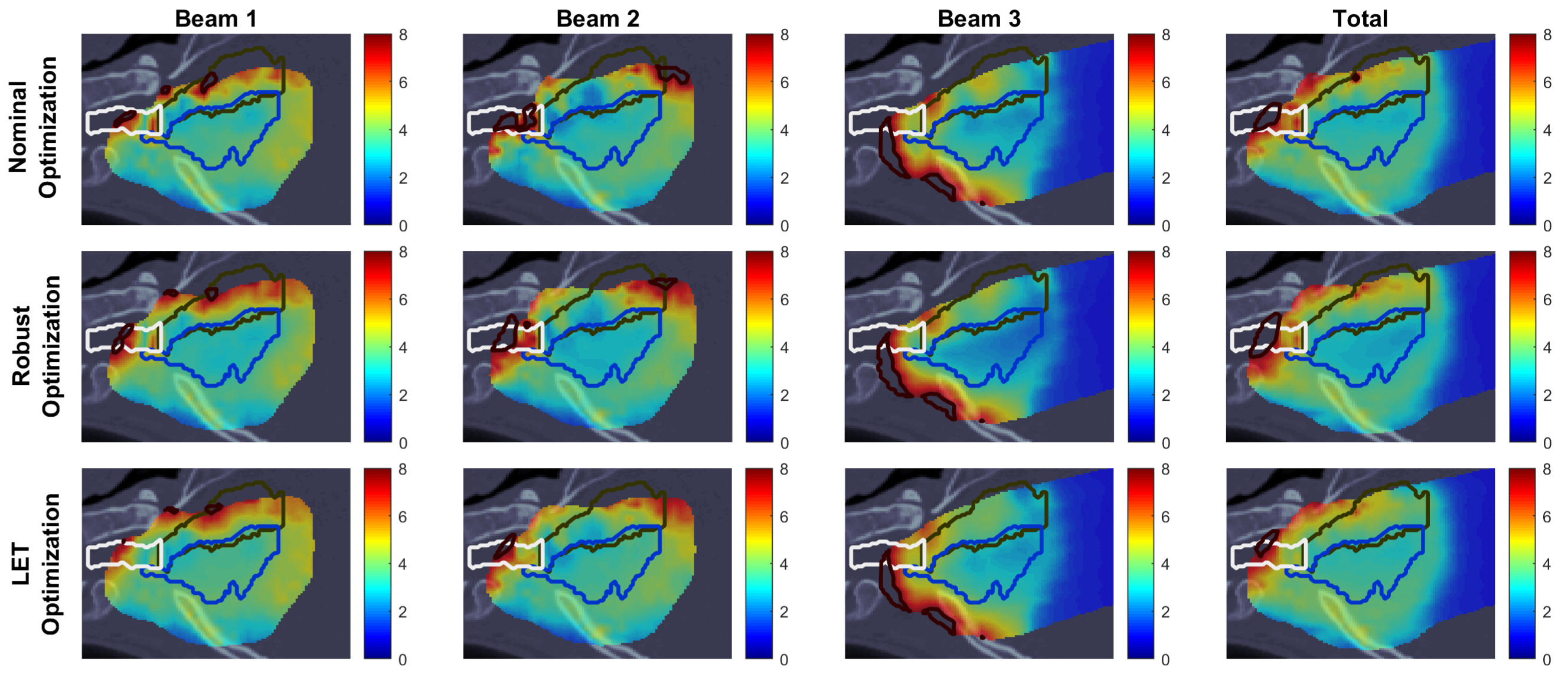


**Figure A2.** Sagittal view of LETd distribution in Case 3. The bright blue contour represents the target region, while the dark blue contour outlines the brainstem. The dark red contour along the beam path indicates regions with higher LETd values that exceed the displayed scale range.


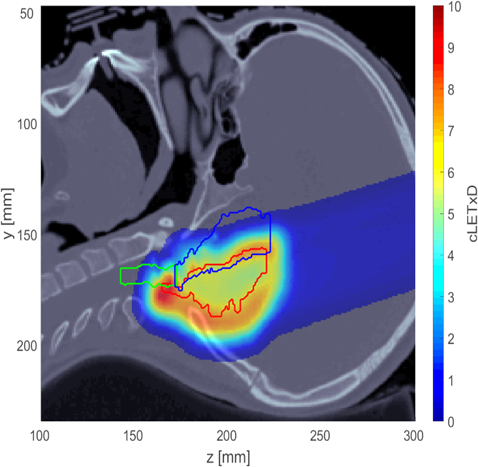

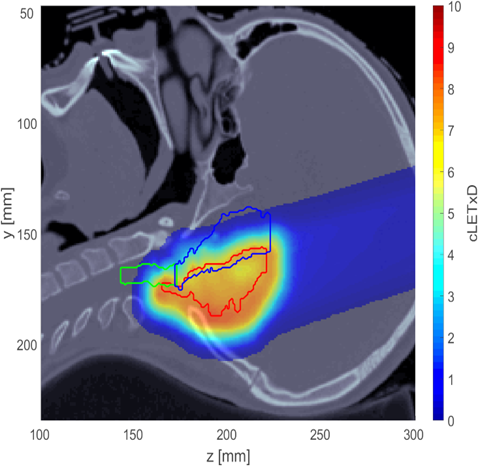


**(a)**

**(b)**

**Figure A3:** Distributions of cLETxD for Case 3: (a) Robust optimization and (b) LET optimization. The red contour delineates the clinical target volume (CTV), the blue contour outlines the brainstem, and the green contour represents the spinal cord. Compared to robust optimization, LET optimization distributes higher cLETxD in the target and lower cLETxD in the spinal cord.

**Table A7.** Worst-case dosimetric [Gy(RBE)] evaluation under uncertainty scenarios for Case 1 across different parameters (rows) and optimization plans (columns). The worst-case max and min represent the highest and lowest dose values observed across 9 uncertainty scenarios. The bandwidth indicates the difference between these extreme values and reflects the plan’s robustness.

| **Parameter** | **Nominal optimization** | | | **Robust optimization** | | | **LET optimization** | | |
| --- | --- | --- | --- | --- | --- | --- | --- | --- | --- |
|  | **Worst-case max** | **Worst-case min** | **Band- width** | **Worst-case max** | **Worst -case min** | **Band-width** | **Worst-case max** | **Worst -case min** | **Band-width** |
| CTV D95% | 56.13 | 49.35 | 6.78 | 55.55 | 51.45 | 4.10 | 56.61 | 50.03 | 6.58 |
| CTV mean | 58.41 | 53.04 | 5.37 | 57.65 | 54.61 | 3.04 | 58.41 | 52.96 | 5.46 |
| CTV max | 70.07 | 59.52 | 10.55 | 66.12 | 60.69 | 5.43 | 63.77 | 59.06 | 4.71 |
| Brainstem mean | 44.76 | 36.58 | 8.18 | 46.25 | 41.45 | 4.80 | 48.05 | 42.21 | 5.84 |
| Brainstem max | 70.07 | 57.58 | 12.49 | 60.59 | 57.14 | 3.45 | 63.66 | 55.71 | 7.95 |
| Spinal cord mean | 4.56 | 1.91 | 2.65 | 6.89 | 3.21 | 3.68 | 7.83 | 3.80 | 4.03 |
| Spinal cord max | 42.56 | 28.77 | 13.80 | 45.48 | 35.41 | 10.07 | 48.79 | 38.21 | 10.58 |

**Table A8.** Worst-case dosimetric [Gy(RBE)] evaluation under uncertainty scenarios for Case 2 across different parameters (rows) and optimization plans (columns). The worst-case max and min represent the highest and lowest dose values observed across 9 uncertainty scenarios. The bandwidth indicates the difference between these extreme values and reflects the plan’s robustness.

| **Parameter** | **Nominal optimization** | | | **Robust optimization** | | | **LET optimization** | | |
| --- | --- | --- | --- | --- | --- | --- | --- | --- | --- |
|  | **Worst-case max** | **Worst -case min** | **Band- width** | **Worst -case max** | **Worst-case min** | **Band-width** | **Worst -case max** | **Worst -case min** | **Band-width** |
| CTV D95% | 57.26 | 49.30 | 7.96 | 54.89 | 49.96 | 4.94 | 57.86 | 48.97 | 8.89 |
| CTV mean | 59.77 | 52.60 | 7.17 | 57.37 | 55.19 | 2.18 | 61.57 | 53.17 | 8.40 |
| CTV max | 64.66 | 56.12 | 8.54 | 61.81 | 58.79 | 3.02 | 65.40 | 56.86 | 8.54 |
| Brainstem mean | 28.32 | 21.58 | 6.74 | 29.86 | 23.14 | 6.72 | 30.46 | 23.67 | 6.79 |
| Brainstem max | 61.95 | 55.65 | 6.31 | 58.76 | 57.58 | 1.18 | 63.05 | 56.54 | 6.51 |
| Spinal cord mean | 3.61 | 2.09 | 1.52 | 4.75 | 3.15 | 1.60 | 4.71 | 3.02 | 1.69 |
| Spinal cord max | 59.57 | 50.62 | 8.95 | 57.65 | 54.40 | 3.25 | 60.15 | 52.04 | 8.11 |

**Table A9.** Worst-case dosimetric [Gy(RBE)] evaluation under uncertainty scenarios for Case 3 across different parameters (rows) and optimization plans (columns). The worst-case max and min represent the highest and lowest dose values observed across 9 uncertainty scenarios. The bandwidth indicates the difference between these extreme values and reflects the plan’s robustness.

| **Parameter** | **Nominal optimization** | | | **Robust optimization** | | | **LET optimization** | | |
| --- | --- | --- | --- | --- | --- | --- | --- | --- | --- |
|  | **Worst -case max** | **Worst -case min** | **Band- width** | **Worst -case max** | **Worst -case min** | **Band-width** | **Worst -case max** | **Worst -case min** | **Band-width** |
| CTV D95% | 56.54 | 48.48 | 8.06 | 56.34 | 50.91 | 5.43 | 57.30 | 49.95 | 7.35 |
| CTV mean | 59.18 | 54.39 | 4.79 | 57.72 | 55.19 | 2.53 | 59.41 | 53.71 | 5.69 |
| CTV max | 66.65 | 59.87 | 6.78 | 62.56 | 58.54 | 4.01 | 64.13 | 58.55 | 5.59 |
| Brainstem mean | 29.01 | 17.47 | 11.54 | 30.97 | 20.4 | 10.57 | 32.09 | 21.04 | 11.05 |
| Brainstem max | 62.33 | 57.69 | 4.64 | 60.09 | 57.01 | 3.08 | 61.08 | 56.1 | 4.98 |
| Spinal cord mean | 18.02 | 9.6 | 8.42 | 17.39 | 11.22 | 6.17 | 20.72 | 12.7 | 8.02 |
| Spinal cord max | 63.64 | 50.35 | 13.3 | 58.14 | 50.55 | 7.59 | 61.52 | 51.54 | 9.97 |

**Table A10.** Worst-case LETd [keV/μm] evaluation under uncertainty scenarios for Case 1 across different parameters (rows) and optimization plans (columns). The worst-case max and min represent the highest and lowest LETd values observed across 9 uncertainty scenarios. The bandwidth indicates the difference between these extreme values and reflects the plan’s robustness.

| **Parameter** | **Nominal optimization** | | | **Robust optimization** | | | **LET optimization** | | |
| --- | --- | --- | --- | --- | --- | --- | --- | --- | --- |
|  | **Worst -case max** | **Worst -case min** | **Band- width** | **Worst -case max** | **Worst -case min** | **Band-width** | **Worst -case max** | **Worst -case min** | **Band-width** |
| CTV mean | 3.73 | 3.41 | 0.32 | 3.32 | 2.99 | 0.32 | 3.65 | 3.37 | 0.28 |
| CTV max | 6.77 | 4.71 | 2.05 | 5.61 | 4.10 | 1.51 | 5.95 | 4.32 | 1.63 |
| Brainstem mean | 4.38 | 4.23 | 0.16 | 3.52 | 3.36 | 0.17 | 3.74 | 3.63 | 0.11 |
| Brainstem max | 9.04 | 6.99 | 2.06 | 5.35 | 4.66 | 0.69 | 5.17 | 4.61 | 0.56 |
| Spinal cord mean | 3.52 | 2.62 | 0.90 | 2.74 | 2.07 | 0.66 | 2.43 | 1.92 | 0.51 |
| Spinal cord max | 12.65 | 11.47 | 1.18 | 10.85 | 10.12 | 0.74 | 9.62 | 9.07 | 0.55 |

**Table A11.** Worst-case LETd [keV/μm] evaluation under uncertainty scenarios for Case 2 across different parameters (rows) and optimization plans (columns). The worst-case max and min represent the highest and lowest LETd values observed across 9 uncertainty scenarios. The bandwidth indicates the difference between these extreme values and reflects the plan’s robustness.

| **Parameter** | **Nominal optimization** | | | **Robust optimization** | | | **LET optimization** | | |
| --- | --- | --- | --- | --- | --- | --- | --- | --- | --- |
|  | **Worst -case max** | **Worst -case min** | **Band- width** | **Worst -case max** | **Worst -case min** | **Band-width** | **Worst -case max** | **Worst -case min** | **Band-width** |
| CTV mean | 3.86 | 3.45 | 0.41 | 3.41 | 2.87 | 0.54 | 4.04 | 3.58 | 0.46 |
| CTV max | 5.12 | 4.39 | 0.73 | 5.01 | 3.76 | 1.24 | 5.05 | 4.25 | 0.80 |
| Brainstem mean | 3.61 | 3.25 | 0.36 | 3.22 | 2.96 | 0.26 | 3.46 | 3.21 | 0.25 |
| Brainstem max | 8.34 | 7.15 | 1.20 | 6.43 | 5.64 | 0.80 | 5.84 | 5.24 | 0.60 |
| Spinal cord mean | 0.59 | 0.48 | 0.11 | 0.56 | 0.49 | 0.08 | 0.59 | 0.50 | 0.10 |
| Spinal cord max | 6.13 | 5.46 | 0.67 | 4.82 | 4.30 | 0.52 | 5.01 | 4.49 | 0.52 |

**Table A12.** Worst-case LETd [keV/μm] evaluation under uncertainty scenarios for Case 3 across different parameters (rows) and optimization plans (columns). The worst-case max and min represent the highest and lowest LETd values observed across 9 uncertainty scenarios. The bandwidth indicates the difference between these extreme values and reflects the plan’s robustness.

| **Parameter** | **Nominal optimization** | | | **Robust optimization** | | | **LET optimization** | | |
| --- | --- | --- | --- | --- | --- | --- | --- | --- | --- |
|  | **Worst -case max** | **Worst -case min** | **Band- width** | **Worst -case max** | **Worst -case min** | **Band-width** | **Worst -case max** | **Worst -case min** | **Band-width** |
| CTV mean | 3.48 | 3.12 | 0.36 | 3.24 | 2.87 | 0.37 | 3.57 | 3.25 | 0.32 |
| CTV max | 7.36 | 5.34 | 2.03 | 6.97 | 5.23 | 1.73 | 6.15 | 4.55 | 1.60 |
| Brainstem mean | 2.61 | 2.07 | 0.54 | 2.48 | 2.18 | 0.31 | 2.41 | 2.18 | 0.23 |
| Brainstem max | 8.18 | 6.40 | 1.77 | 6.62 | 5.38 | 1.24 | 6.18 | 5.06 | 1.12 |
| Spinal cord mean | 3.11 | 2.14 | 0.97 | 2.84 | 2.29 | 0.54 | 2.27 | 1.96 | 0.32 |
| Spinal cord max | 10.83 | 8.87 | 1.96 | 9.82 | 8.19 | 1.62 | 7.58 | 6.30 | 1.28 |
